# Screen Time on School Days and Risks for Psychiatric Symptoms and Self-Harm in Mainland Chinese Adolescents

**DOI:** 10.3389/fpsyg.2016.00574

**Published:** 2016-04-25

**Authors:** Mingli Liu, Qingsen Ming, Jinyao Yi, Xiang Wang, Shuqiao Yao

**Affiliations:** ^1^Medical Psychological Institute, Second Xiangya Hospital of Central South UniversityChangsha, China; ^2^School of Education, Hunan University of Science and TechnologyXiangtan, China

**Keywords:** sedentary behavior, physical activity, depression, anxiety, attention deficit/hyperactivity, oppositional defiant, conduct problems, suicide

## Abstract

**Objective:** To investigate associations of television and of video game or non-educational computer use (VG/CU) exposure volumes in a typical school day with psychiatric symptoms and suicidal ideation/self-injurious behavior (self-harm), in mainland Chinese adolescents.

**Methods:** Secondary school pupils (*N* = 13,659; mean age: 15.18 ± 1.89) from 10 urban areas sampled from different regions of mainland China were recruited. The subjects were divided into the following four screen exposure volume groups for television and VG/CU respectively based on a self-administered questionnaire: 0 h/day, >0 to ≤1 h/day, >1 to ≤2 h/day, and >2 h/day. Demographic and psychiatric symptoms were recorded for each respondent. Odds ratios (ORs) and 95% confidence intervals (CIs) for several types of psychological problems and self-harm were calculated.

**Results:** More than 2 h per school day television watching was associated with higher risk of depression in both boys (OR = 1.33, 95%CI: 1.02–1.73) and girls (OR = 1.62, 95%CI: 1.19–2.21), of anxiety in boys (OR = 1.43, 95%CI: 1.05–1.95), of general emotional, behavioral, and social problems (GEBSPs; OR = 1.55, 95%CI: 1.01–2.39), and of oppositional defiant problems (OR = 1.65, 95% CI: 1.09–2.50) in girls, compared with no television exposure. Conversely, television exposure of no more than 1 h per school day was associated with lower self-harm risk in boys (OR = 0.81, 95%CI: 0.67–0.99) compared with no television exposure. High school day VG/CU time (>2 h) compared with no VG/CU were associated with higher risks of anxiety (OR = 1.40, 95%CI: 1.06–1.86) and of attention deficit/hyperactivity problems (ADHPs; OR = 1.56, 95%CI: 1.02–2.38) in boys. And any school day VG/CU exposure was associated with higher risks of self-harm and all other psychiatric problems in boys and all psychiatric problems (including anxiety and ADHPs) in girls (ORs, 1.44–3.69), compared to no VG/CU exposure.

**Conclusion:** For secondary school students, associations of psychiatric problems and self-harm were more strongly associated with exposure to VG/CU than with exposure to television. The findings suggest that VG/CU and television exposure on weekdays should be considered in psychiatric interventions for adolescents.

## Introduction

Psychological health problems are a primary cause of health-related burdens in adolescents, influencing 10–20% of this age group worldwide (Kieling et al., 2011). Adolescence is a critical period of health development, and poor psychological health during this period is associated with numerous problems, such as low academic achievement, substance abuse, violence, and even suicide (Patel et al., 2007). Furthermore, most psychological problems are long-lasting and will continue to impact affected individuals into adulthood (73.9–77.9% adults cases defined by diagnostic interview schedule or clinically diagnosed had received a diagnosis before 18 years of age and 50.0–77.9% before 15 years of age; Kim-Cohen et al., 2003; Haarasilta et al., 2004; Kessler et al., 2007; Kieling et al., 2011). Psychological health risk and protective factors must be better understood to reduce the burden of psychological problems.

Extensive exposure to electronic media has been suggested as an “invisible” risk factor for psychiatric symptoms and suicidal behavior (Carli et al., 2014). Today’s adolescents are growing up with increased access to both old and new forms media and using them in various ways (Strasburger et al., 2010; Blum et al., 2012). Time spent consuming and using electronic devices increases markedly with economic and technological improvements. Indeed, given that today’s juveniles often spend more time on screen than they do engaging in any other behaviors (Rideout et al., 2010), with the exception of sleep, it is very important to explore the psychological effects of ST, particularly in relation to the sheer volume of ST exposure.

The most frequent forms of ST that adolescents engage in are television watching and computer use (Rideout et al., 2010). Some have suggested that ST may have potentially positive effects. For example, according to social learning theory, children may learn social lessons by observing and imitating what they see on screen (Bandura, 2001). Conversely, the displacement hypothesis (Kraut et al., 1998) posits that time spent using electronic media displaces time that could be spent on productive and/or active activities, as well as time spent sleeping (Primack et al., 2009), which may impede normal cognitive and emotional development (Van den Bulck, 2004; Eggermont and Van den Bulck, 2006). Additionally, the content of the media adolescents are exposed to may influence their psychological health. To maximize the benefits and minimize the negative effects of ST on juveniles’ mental healthy development, we need to conduct in-depth studies exploring how ST, especially volume of ST exposure, influences adolescents. It is important to determine what amount of ST may be beneficial, if any, and to determine at what volume ST becomes harmful. Elucidation of such information will allow guidelines based on empirical research to be made available to parents, educators, and pediatricians internationally.

Studies investigating associations between ST and psychological health in adolescence have yielded contradictory findings. For example, some studies have suggested that greater ST was associated with a higher risk of depression (Primack et al., 2009; Desai et al., 2010), whereas others reported the opposite relationship (Chen and Lu, 2009; Casiano et al., 2012) or no association (McHale et al., 2001). Most studies in this area have examined whether ST is associated with particular mental health problems using the pediatricians’ ST recommendation of no more than 2 h per day as a threshold level (Pediatrics, 2001), which is based largely on narrative reviews and expert opinions (Tremblay et al., 2011). Based on this approach, researchers have suggested a linear association between ST and mental health problems. However, there is limited information about ST quantity and particular psychiatric problems. Also, most previous studies have used an average daily ST parameter that averages weekday and weekend use. Averaging weekday and weekend use is problematic because adolescents tend to spend much more time with electronic media on weekend days than on weekdays (Biddle et al., 2009; Hamar et al., 2010).

Recent studies examining multiple ST exposure levels have suggested a non-linear association between ST and mental health (Hong et al., 2009; Bélanger et al., 2011; Messias et al., 2011; Kim, 2012; Liu et al., 2015b). That is, these studies have suggested that ST in limited amounts was associated with a lower risk of mental problems relative to no ST. However, these studies involved primarily children in developed countries; nationally investigations in developing countries are lacking.

Here, we examined the associations of tiered volumes of television watching and of playing video or computer games or using a computer for non-education purpose (VG/CU) in a typical school day with multiple psychiatric symptoms and suicidal ideation/self-injurious behavior (“self-harm” for simplicity from here forward), compared with no television or VG/CU exposure, in a multicenter, population-based mainland Chinese adolescent cohort. We hypothesized that television and VG/CU would have different associations with psychiatric problems and self-harm, and further hypothesized that these associations would vary by gender and in relation to ST volume.

## Materials and Methods

### Participants

Secondary school pupils (*N* = 13,659; mean age: 15.18 ± 1.89) from 10 urban areas (Beijing, Shanghai, Shenyang, Hangzhou, Suzhou, Changsha, Chengdu, Guangzhou, Yinchuan, and Langfang) across mainland China were recruited. A multi-stage cluster sampling method was employed to produce a representative sample of the sampled cities. Firstly, based on academic performance, schools in each city were categorized into three types: high, medium, and low. Then, two or three medium-level schools in each city were selected randomly. One, two, or three classes in each grade (7th through 12th grade) of each selected school (total 23 schools) were then selected randomly. Before the survey, all students and their parents provided written informed consent to participate in this study, which was approved by the Human Subjects Review Committee at the Second Xiangya Hospital of Central South University.

### Questionnaires

A self-report questionnaire packet was administered to participating adolescents. The packet included: a socio-demographic questionnaire that collected personal information such as age, gender, school, grade, height, weight, and socioeconomic status; the Youth Risk Behavior Survey (YRBS) questionnaire (Brener et al., 2002), which assessed exposure volumes for television, VG/CU, physical activity, and smoking; the Centers for Epidemiologic Studies-Depression Scale (CES-D; Wang et al., 2013); the Multidimensional Anxiety Scale for Children (MASC; March et al., 1997; Yao et al., 2007); the Youth Self-Report (YSR; Achenbach et al., 2001; Yao et al., 2009); and the self-harm subscale of the Health-Risk Behavior Inventory for Chinese Adolescents (HBICA; Wang et al., 2012). All of the scales and subscales used were confirmed to be internally consistent (Cronbach’s alpha range, 0.64–0.95).

Two items from the YRBS questionnaire were used as the measures of the amount of ST. Respondents were asked to report “How many hours do you watch television or play VG/CU (including activities such as Nintendo, Game boy, Xbox, computer games, and the internet) on a typical school day?” The possible responses to the question include the following categories: 0 h, less than 1, 1, 2, 3, 4, 5, or more hours. Based on previous studies’ findings, especially on a recent meta-analysis conducted by Liu et al. (2015b) which suggested a non-linear association between ST and depression at ST <2 h/day, with the lowest risk being detected at ST of 1 h/day, we grouped these responses into four categories: non-ST (0 h/day), occasional ST (>0 to ≤1 h/day), moderate ST (>1 to ≤2 h/day), and high ST (>2 h/day). Socio-demographic, psychiatric, and self-harm assessment results were stratified by screen exposure volume.

We scaled cutoff scores to stratify adolescents into dichotomous psychiatric categories as follows: depression [CES-D score ≥ 22 for males and ≥ 24 for females (Roberts et al., 1991; Primack et al., 2009)]; anxiety (MASC *t*-score ≥ 65); General emotional, behavioral, and social problems (GEBSPs; The total score of YSR, above the 90th percentile); ADHPs [YSR-ADHP diagnostic subscale *t*-score ≥ 65 (Achenbach and Rescorla, 2001; Achenbach et al., 2001)]; ODPs [YSR-ODPs diagnostic subscale *t*-score ≥ 65 (Achenbach and Rescorla, 2001; Achenbach et al., 2001)]; CPs [YSR-CPs diagnostic subscale *t*-score ≥ 65 (Achenbach and Rescorla, 2001; Achenbach et al., 2001)]; and self-harm [HBICA subscale, above the 90th percentile (Wang et al., 2012)]. All cutoffs based on percentile or *t*-score were calculated separately by gender.

### Statistical Analysis

We used *F*-tests for continuous variables and chi-square tests for categorical variables to compare the ST distributions in a typical school day across socio-demographic, psychiatric symptoms, and self-harm groups. Meanwhile, effect sizes (η^2^ for ANOVA and φ or Cramer v for Chi-square tests) were estimated. All analyses were further stratified by screen type (television and VG/CU).

According to previous studies, some other variables, such as sociodemographic variables, may be associated with juveniles’ ST and could affect their mental health (Shapira et al., 2000; Kim et al., 2006). Therefore, in order to assess the associations of ST with psychiatric symptoms and self-harm behavior after adjusting for potential confounding effects, separate multivariate logistic regression analyses using television and VG/CU time respectively as an independent variable and each psychiatric symptom as a dependent variable were performed. The covariates included age, grade, BMI, subjective economic status, physical activity, smoking, and television time for VG/CU time or vice versa. The analyses were stratified by gender. Odds ratios (ORs) and corresponding 95% confidence intervals (CIs) were used as measures of group associations, with the no television or VG/CU exposure groups serving as the reference groups. The tests for trend in risks with increasing ST order which were determined by logistic regression models were performed. For all analyses, we used *p* < 0.05 (two-tailed) to define statistical significance. Mean values are reported with standard deviation (SD). The statistical analyses were performed in SPSS 19.0 and STATA 12.0 software (Stata Corp., College Station, TX, USA).

## Results

### Characteristics of Participants and ST

Adolescents’ characteristics involving various exposure volumes of screen were heterogeneous. The characteristics of participants in each exposure volume group, stratified by screen type, are reported in **Table 1**.

**Table 1:** Characteristics of participants by ST exposure level**^†^**.

Variables	Total (*N* = 13414)	Television time (h/school day)					VG/CU time (h/school day)				
		0 (*N* = 7866; 58.6%)	>0 to ≤1 (*N* = 3894; 29.1%)	>1 to ≤2 (*N* = 935; 7.0%)	>2 (*N* = 710; 5.3%)	*ES; p*	0 (*N* = 9288; 70.2%)	>0 to ≤1 (*N* = 2434; 18.4%)	>1 to ≤2 (*N* = 761; 5.8%)	>2 (*N* = 736; 5.6%)	*ES; p*
Female gender, *N* (%)	6598 (49.3)	4244 (54.2)	1691 (43.4)	388 (41.6)	275 (38.7)	0.12; <0.001	4856 (52.5)	1111 (45.6)	319 (42.1)	240 (32.7)	0.11; <0.001
Age, mean (SD)	15.2 (1.9)	15.3 (1.9)	15.1 (1.85)	14.8 (1.8)	14.9 (1.8)	0.01; <0.001	15.2 (1.9)	15.2 (1.8)	15.1 (1.8)	15.1 (1.7)	0.00; 0.077
Junior grade^‡^, *N* (%)	6732 (50.2)	3706 (47.1)	2059 (52.8)	574 (61.4)	393 (55.4)	0.08; <0.001	4599 (49.5)	1180 (48.4)	394 (51.8)	373 (50.7)	0.02; 0.361
BMI, mean (SD)	19.6 (3.4)	19.5 (3.2)	19.8 (3.5)	19.6 (3.6)	19.6 (3.7)	0.00; 0.007	19.6 (3.3)	19.7 (3.4)	19.7 (3.5)	19.7 (3.7)	0.00; 0.392
SES^¶^, mean (SD)	6.1 (1.6)	6.1 (1.6)	6.2 (1.6)	6.0 (1.6)	6.0 (1.8)	0.00; 0.026	6.1 (1.6)	6.1 (1.6)	6.0 (1.7)	6.1 (1.7)	0.00; 0.429
Physical activity, *N* (%)							
0 days/week	5465 (40.9)	3542 (45.2)	1363 (35.0)	303 (32.5)	257 (36.4)	0.09; <0.001	3920 (42.4)	923 (38.0)	278 (36.6)	294 (40.2)	0.04; <0.001
1–3 days/week	5275 (39.4)	2864 (36.5)	1739 (44.7)	432 (46.3)	240 (33.9)		3553 (38.4)	1064 (43.8)	329 (43.3)	252 (34.5)	
4–7 days/week	2633 (19.7)	1438 (18.3)	787 (20.2)	198 (21.2)	210 (29.7)		1783 (19.3)	445 (18.3)	153 (20.1)	185 (25.3)	
Ever smoke, *N* (%)	969 (7.3)	422 (5.4)	317 (2.4)	109 (11.8)	121 (17.2)	0.12; <0.001	500 (5.4)	244 (10.1)	102 (13.5)	110 (15.1)	0.12; <0.001

*ST, screen time; VG/CU, playing video or computer games or using a computer for non-educational purpose; ES, effect size, η^2^ for ANOVA, φ or Cramer v for Chi-square test; BMI, body mass index; SES, subjective economic status; ^†^Percentages do not always equal 100 because of rounding. Values may not always sum to sample size because of missing data; ^‡^Junior grade indicates 7th–9th grade; ^¶^ SES was considered as continuous variables (ranging from 1-the worst, to 10-the best).*

Nearly half (49.3%) of the participants were female, and slightly more than half (50.2%) were in junior high school. Their mean age (SD) was 15.2 (1.9) years, and their mean BMI (SD) was 19.6 (3.4). Their mean subjective social and economic status score (SD) was 6.1 (1.6; range, 1-the lowest to 10-the highest). Only 19.7% of the subjects reported engaging in moderate to vigorous physical activity (≥60 cumulative minutes per day) at least 4 days per week, while 40.9 and 39.4% reported 0 or 1–3 physically active days per week, respectively. Most of the participants (92.7%) reported never smoking; only 7.3% reported having ever smoked.

More than half of the participants (58.6%) reported never watching television on school days, 29.1% reported spending no more than 1 h, and 5.3% reported spending more than 2 h. Television exposure volume differed significantly in relation to socio-demographic variables, physical activity, and smoking. However, the effect sizes for the differences were small (0.00 to 0.12).

A substantial majority (70.2%) of the adolescent participants reported no VG/CU on a typical school day, 18.4% reported spending no more than 1 h, and 5.6% reported spending more than 2 h. Gender (*p* < 0.001), physical activity (*p* < 0.001), and smoking (*p* < 0.001) were significant factors associated with VG/CU exposure volume. Likewise, the effect sizes for these differences were small (0.04 to 0.12).

### Psychiatric Symptoms and Self-Harm Behavior

The prevalence rates for examined psychiatric symptoms and self-harm behavior (based on psychometric cutoffs) in our adolescent cohort, stratified by screen type and exposure, are reported in **Table 2**. Overall, 27.3 and 7.4% of our adolescent subjects showed symptoms of depression and anxiety, respectively. GEBSPs were detected in 10.2% of the participants. Meanwhile, 6.4% of adolescents had ADHPs, 11.1% had ODPs (including >3% with a *t*-score between 64.5 and 65), and 7.3% had CPs. Furthermore, 10.8% of the participants reported self-harm issues.

**Table 2:** Prevalence (%)**^†^** of psychiatric symptoms by ST exposure levels.

Variables	Total *N* (%)	Television time (h/school day) *N* (%)					VG/CU time (h/school day) *N* (%)				
		0	>0 to ≤1	>1 to ≤2	>2	ES; *p*	0	>0 to ≤1	>1 to ≤2	>2	ES;*p*
Depression, *N* (%)	3499 (27.3)	1996 (26.2)	1013 (26.7)	283 (31.2)	251 (36.8)	0.06; <0.001	2228 (24.7)	736 (31.2)	258 (35.2)	277 (39.2)	0.10; <0.001
Anxiety, *N* (%)	944 (7.4)	538 (7.2)	259 (7.0)	71 (8.0)	76 (11.5)	0.04; <0.001	603 (6.8)	179 (7.8)	78 (10.8)	73 (10.8)	0.05; <0.001
General emotional, behavioral, and social problems, *N* (%)	1249 (10.2)	709 (9.6)	365 (10.0)	100 (12.3)	94 (16.3)	0.05; <0.001	754 (8.7)	273 (12.1)	97 (14.3)	125 (19.7)	0.09; <0.001
Attention deficit/hyperactivity problems, *N* (%)	769 (6.4)	420 (5.9)	233 (6.6)	64 (8.3)	52 (9.6)	0.04; 0.001	464 (5.5)	165 (7.7)	65 (10.1)	66 (11.0)	0.07; <0.001
Oppositional defiant problems, *N* (%)	1355 (11.1)^‡^	717 (9.8)	418 (11.6)	112 (14.1)	108 (19.4)	0.07; <0.001	795 (9.3)	301 (13.6)	110 (16.6)	133 (21.5)	0.10; <0.001
Conduct problems, *N* (%)	871 (7.3)	451 (6.4)	268 (7.7)	73 (9.5)	79 (14.5)	0.07; <0.001	479 (5.8)	187 (8.6)	88 (13.6)	103 (17.4)	0.12; <0.001
Suicide and self-injury, *N* (%)	1430 (10.8)	802 (10.4)	407 (10.6)	113 (12.3)	108 (15.5)	0.04; <0.001	840 (9.2)	321 (13.3)	117 (15.7)	135 (18.9)	0.09; <0.001

*ST, screen time; VG/CU, playing video or computer games or using a computer for non-educational purpose; ^†^Percentages do not always equal 100 because of rounding. Values may not always sum to sample size because of missing data; ^‡^total prevalence of oppositional defiant problems was 11.1% for more than 3% samples’ T score was between 64.5 to 65 (cutoff: t-score ≥ 64.50).*

The risk prevalence values for all assessed psychiatric symptoms and self-harm behavior differed across ST exposure levels (*p* ≤ 0.001). Anxiety prevalence was lowest (7.0%) among subjects who watched television >0 to ≤1 h per school day. For self-harm behavior and all other types of psychiatric problems (including anxiety in VG/CU time), prevalence rates were lowest in the no screen exposure per school day group, with prevalence rates increasing with greater ST. The effect sizes for the differences of the risk prevalence in different ST exposure groups were ranged from 0.04 to 0.12.

### Associations of ST with Psychiatric Symptoms and Self-Harm

**Table 3** presents the correlations coefficients between all key variables in the analyses (male and female). All the correlation coefficients reached significant levels with the exception of the correlation between television time and anxiety. The correlation coefficients between ST time and each psychiatric symptom range from 0.044 to 0.126 in television and 0.024 to 0.180 in VG/CU.

**Table 3:** Correlations matrix between the measurement variables.

	TV time	VG/CU time	Depression	Anxiety	GEBSPs	ADHPs	ODs	CPs	Self-harm
TV time	—								
VG/CU time	0.391^∗∗∗^	—							
Depression	0.05^∗∗∗^	0.103^∗∗∗^	—						
Anxiety	-0.001	0.024^∗∗^	0.532^∗∗∗^	—					
GEBSPs	0.046^∗∗∗^	0.106^∗∗∗^	0.558^∗∗∗^	0.530^∗∗∗^	—				
ADHPs	0.065^∗∗∗^	0.112^∗∗∗^	0.409^∗∗∗^	0.348^∗∗∗^	0.727^∗∗∗^	—			
ODs	0.097^∗∗∗^	0.143^∗∗∗^	0.382^∗∗∗^	0.293^∗∗∗^	0.682^∗∗∗^	0.589^∗∗∗^	—		
CPs	0.126^∗∗∗^	0.180^∗∗∗^	0.329^∗∗∗^	0.227^∗∗∗^	0.674^∗∗∗^	0.498^∗∗∗^	0.542^∗∗∗^	—	
Self-harm	0.044^∗∗∗^	0.101^∗∗∗^	0.433^∗∗∗^	0.281^∗∗∗^	0.453^∗∗∗^	0.280^∗∗∗^	0.304^∗∗∗^	0.350^∗∗∗^	—

*VG/CU, playing video or computer games or using a computer for non-educational purpose; GEBSPs, general emotional, behavioral, and social problems; ADHPs, attention deficit/hyperactivity problems; ODs, oppositional defiant problems; CPs, conduct problems; ^∗∗^p < 0.01, ^∗∗∗^p < 0.001.*

The logistic regression analysis results (adjusted ORs and 95%CIs) for each psychiatric symptom and self-harm in relation to television exposure volume are reported, stratified by gender, in **Table 4**. On the whole, adjusted for potential confounding variables, television time was not significantly associated with self-harm or with most of the accessed psychiatric problems in either males or females. However, >2 h per school day of television watching was associated with higher risk of depression in both genders (males, OR = 1.33, 95%CI: 1.02–1.73; females, OR = 1.62, 95%CI: 1.19–2.21), higher risk of anxiety in males (OR = 1.43, 95%CI: 1.05–1.95), and higher risk of GEBSPs (OR = 1.55, 95%CI: 1.01–2.39) and of ODPs in females (OR = 1.65, 95%CI: 1.09–2.50), compared with the reference group. And only depression and ODPs increased with increasing level of television time in female (*p* < 0.05 for Trend). Meanwhile, television watching in moderation (>0 to ≤1 h per school day) was associated with lower self-harm risk in males compared with the no television reference group (OR = 0.81, 95%CI: 0.67–0.99). We further performed *post hoc* comparisons for three non-zero television groups, however, no significant difference was found.

**Table 4:** Logistic regression analysis of associations between psychiatric symptoms, self-harm and television ST for each gender.

Outcomes	Self-reported television exposure levels (h/school day)						
	Males				Females			
	>0 to ≤1	>1 to ≤2	>2	*p*-value for trend	>0 to ≤1	>1 to ≤2	>2	*p*-value for trend
Depression, OR (95%CI), *p*	0.92 (0.80 to 1.05) 0.231	1.03 (0.82 to 1.30) 0.804	1.33 (1.02 to 1.73) 0.032	0.218	1.00 (0.86 to 1.16) 0.979	1.10 (0.84 to 1.44) 0.485	1.62 (1.19 to 2.21) 0.002	0.013
Anxiety, OR (95%CI), *p*	0.88 (0.74 to 1.05) 0.167	1.14 (0.86 to 1.51) 0.374	1.43 (1.05 to 1.95) 0.024	0.112	0.94 (0.73 to 1.21) 0.627	0.87 (0.55 to 1.36) 0.533	1.51 (0.96 to 2.38) 0.072	0.366
General emotional, behavioral, and social problems, OR (95%CI), *p*	0.93 (0.76 to 1.15) 0.518	1.04 (0.73 to 1.48) 0.840	1.33 (0.89 to 1.97) 0.160	0.325	0.89 (0.71 to 1.12) 0.326	0.97 (0.64 to 1.47) 0.889	1.55 (1.01 to 2.39) 0.045	0.210
Attention deficit/hyperactivity problems, OR (95%CI), *p*	1.06 (0.82 to 1.37) 0.643	1.41 (0.94 to 2.11) 0.101	0.97 (0.56 to 1.67) 0.903	0.473	0.96 (0.73 to 1.27) 0.775	1.03 (0.62 to 1.72) 0.908	1.48 (0.86 to 2.53) 0.153	0.326
Oppositional defiant problems, OR (95%CI), *p*	0.93 (0.77 to 1.13) 0.480	1.23 (0.90 to 1.70) 0.196	1.34 (0.92 to 1.94) 0.127	0.543	1.04 (0.83 to 1.29) 0.747	1.14 (0.77 to 1.68) 0.510	1.65 (1.09 to 2.50) 0.019	0.031
Conduct problems, OR (95%CI), *p*	0.84 (0.64 to 1.09) 0.187	1.03 (0.69 to 1.55) 0.869	1.34 (0.86 to 2.09) 0.192	0.679	1.14 (0.89 to 1.47) 0.307	0.86 (0.52 to 1.41) 0.546	1.45 (0.89 to 2.38) 0.137	0.216
Self-harm, OR (95%CI), *p*	0.81 (0.67 to 0.99) 0.037	0.81 (0.58 to 1.12) 0.206	0.89 (0.62 to 1.27) 0.510	0.464	0.95 (0.77 to 1.17) 0.620	0.80 (0.54 to 1.18) 0.258	1.26 (0.85 to 1.88) 0.250	0.740

*ST, screen time; OR, adjusted odds ratio; CI, confidence interval; adjusted variables: age, grade, body mass index, subjective economic status, volume of video games/computer use for non-education purpose, smoking, physical activity; gender-matched no television exposure groups served as reference groups.*

The logistic regression analysis results (adjusted ORs and 95%CIs) for each psychiatric symptom and self-harm in relation to VG/CU exposure volume are reported, stratified by gender, in **Table 5**. After controlling for confounding variables, VG/CU time of more than 2 h per school day was found to be associated with a higher risk of anxiety (OR = 1.40, 95%CI: 1.06–1.86) and a higher risk of ADHPs (OR = 1.56, 95%CI: 1.02–2.38) in males, compared with the no VG/CU exposure reference group. And ADHPs increased with increasing level of VG/CU time (*p* < 0.05 for trend) in male. Daily higher VG/CU exposure was associated with a higher risk of self-harm and with all other psychiatric problems in both genders (including anxiety and ADHPs in females) with a stronger trend (*p* < 0.001 for trend), compared with the no screen exposure reference group.

**Table 5:** Logistic regression analysis of associations between psychiatric symptoms, self-harm and VG/CU exposure for each gender.

Outcomes	Self-reported VG/CU exposure levels (h/school day)						
	Males				Females			
	>0 to ≤1	>1 to ≤2	>2	*p*-value for trend	>0 to ≤1	>1 to ≤2	>2	*p*-value for trend
Depression, OR (95%CI), *p*	1.34 (1.15 to 1.56) <0.001	1.39 (1.09 to 1.77) 0.008	1.61 (1.28 to 2.03) <0.001	<0.001	1.44 (1.21 to 1.70) <0.001	2.15 (1.63 to 2.82) <0.001	1.96 (1.42 to 2.71) <0.001	<0.001
Anxiety, OR (95%CI), *p*	1.11 (0.91 to 1.36) 0.284	1.10 (0.81 to 1.50) 0.553	1.40 (1.06 to 1.86) 0.018	0.122	1.56 (1.19 to 2.05) 0.001	2.45 (1.65 to 3.62) <0.001	1.78 (1.09 to 2.89) 0.021	<0.001
General emotional, behavioral, and social problems, OR (95%CI), *p*	1.46 (1.16 to 1.84) 0.001	1.60 (1.12 to 2.28) 0.010	2.18 (1.58 to 3.02) <0.001	<0.001	1.68 (1.32 to 2.14) <0.001	2.04 (1.39 to 2.98) <0.001	3.69 (2.48 to 5.51) <0.001	<0.001
Attention deficit/hyperactivity problems, OR (95%CI), *p*	1.19 (0.89 to 1.59) 0.229	1.47 (0.96 to 2.26) 0.080	1.56 (1.02 to 2.38) 0.039	0.012	1.74 (1.29 to 2.34) <0.001	2.13 (1.34 to 3.38) 0.001	3.17 (1.94 to 5.17) <0.001	<0.001
Oppositional defiant problems, OR (95%CI), *p*	1.38 (1.11 to 1.71) 0.004	1.56 (1.12 to 2.18) 0.009	1.87 (1.36 to 2.56) <0.001	<0.001	1.75 (1.39 to 2.20) <0.001	1.88 (1.30 to 2.74) 0.001	3.59 (2.43 to 5.30) <0.001	<0.001
Conduct problems, OR (95%CI), *p*	1.71 (1.28 to 2.29) <0.001	2.97 (2.02 to 4.35) <0.001	3.41 (2.36 to 4.93) <0.001	<0.001	1.48 (1.12 to 1.97) 0.006	2.16 (1.42 to 3.29) <0.001	3.25 (2.06 to 5.13) <0.001	<0.001
Self-harm, OR (95%CI), *p*	1.56 (1.26 to 1.93) <0.001	1.68 (1.22 to 2.31) 0.002	1.82 (1.37 to 2.48) <0.001	<0.001	1.58 (1.26 to 1.99) <0.001	2.17 (1.52 to 3.10) <0.001	3.10 (2.13 to 4.52) <0.001	<0.001

*VG/CU, playing video or computer games or using a computer for non-educational purpose; OR, adjusted odds ratio; CI, confidence interval; adjusted variables: gender, age, grade, body mass index, subjective economic status, volume of television time, physical activity, smoking; gender-matched no VG/CU exposure groups served as the reference group.*

**FIGURE 1:**
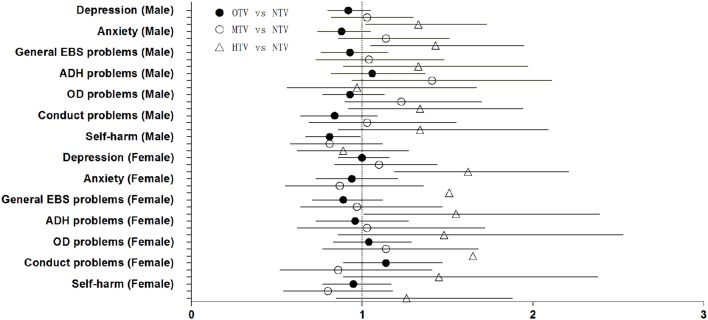
**Forest plot of the association between psychiatric sympotoms, self-harm and school day television time (h/day).** Logistic regression results (Detailed odds ratios and corresponding 95% confidence intervals are given in **Table 4**); NTV, non-television; OTV, occasional television (>0 to ≤1 h/day); MTV, moderate television (>1 to ≤2 h/day); HTV, high television (>2 h/day); General EBS problems, genera emtional, behavioral, and social problems; ADH problems, attention deficit/hyperactivity problems; OD problems, oppositional defiant problems.

**FIGURE 2:**
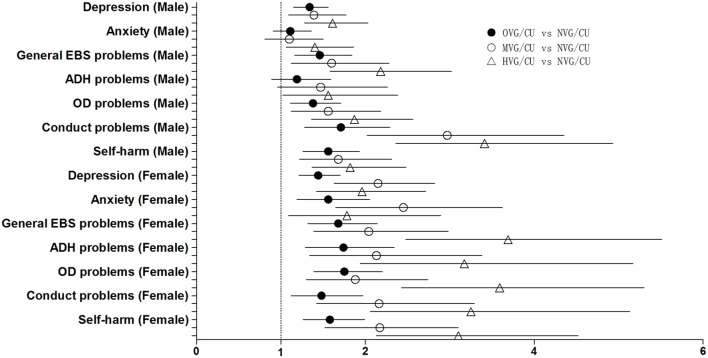
**Forest plot of the association between psychiatric sympotoms, self-harm and school day VG/CU time (h/day).** Logistic regression results (Detailed odds ratios and corresponding 95% confidence intervals are given in **Table 5**); VG/CU, playing video or computer games or using a computer for non-educational purpose, General EBS problems, genera emtional, behavioral, and social problems, ADH problems, attention deficit/hyperactivity problems, OD problems, oppositional defiant problems; NVG/CU, non-VG/CU; OVG/CU, occasional VG/CU (>0 to ≤1 h/day); MVG/CU, moderate VG/CU (>1 to ≤2 h/day); HVG/CU, high VG/CU (>2 h/day).

**Figures 1** and **2** displays the relevant results of the associations between psychiatric symptoms, self-harm and television and VG/CU time respectively in a single plot (including both genders). Three types of dot represent the point estimates of the regression, and horizontal lines depict 95%CIs. The vertical dotted line at 1 point (representing the null hypothesis) is to detect whether an Odds Ratio reaches a statistically significant level (when the confidence interval crossing the vertical line at 1 point). **Figure 1** shows that there are only five psychiatric symptoms’ confidence intervals are crossing the dotted line being in stark contrast to almost all of the symptoms in **Figure 2**. A comparison between the two figures shows that associations of psychiatric problems and self-harm are much more strongly associated with exposure to VG/CU than with exposure to television.

## Discussion

The current study indicated that time spent watching television and time spent engaging in VG/CU in a typical weekday have distinct associations with psychiatric problems and self-harm in mainland Chinese adolescents. On the whole, VG/CU time had stronger associations than television ST with psychiatric problems and self-harm. The results support pediatricians’ recommendations in developed countries concerning limiting ST.

Our findings that >2 h per weekday of television ST was associated with symptoms of depression and anxiety in male adolescents, and associated with depression, GEBSPs, and ODPs in female adolescents, relative to no television ST, are in line with Primack et al.’s (2009) study involving US adolescents. In that study, Primack et al. (2009) reported a link between television ST exposure (weekday and weekend) and subsequent depression (OR = 1.08, 95%CI: 1.01–1.16). These results support the hypothesis that excessive media exposure may displace other protective experiences, such as productive/active activities and sleep.

Interestingly, we found that limited television watching (>0 to ≤1 h/weekday) was associated with lower risk of self-harm in males. This finding is partially consistent with previous work (Bélanger et al., 2011; Kim, 2012) suggesting a U-shaped non-linear association between ST (computer/internet exposure on weekdays and weekends in the prior studies) and depression, perceived stress, and suicidal ideation. Hence, the supposition that media, in moderation, could have potentially positive impacts on adolescents, such as social modeling, is supported by this result (Bandura, 2001). It is not known why this protective effect was significant for boys only. However, it could be that boys benefitted especially from the ST if it was experienced together with other families members. Children co-viewing television with their parents has been associated with stronger family connectedness (Padilla-Walker et al., 2012; Coyne et al., 2014), and family connectedness may be a particularly salient protective factor in boys. Family connectedness stood alone as a primary protective factor against suicide in boys in a large national US study, but was a co-primary protective factor with emotional wellbeing in girls (Borowsky et al., 2001). However, there was no sign of U-shaped non-linear association between television watching and boys’ self-harm in this study. Actually, no significant difference was found among three non-zero television groups. Besides, the association between low television watching and self-harm in boys, compared with no television exposure, appears to be weak (*p* = 0.037). And it is no longer significant (*p* = 0.111) after adjusting p-value by Holm’s procedure. Therefore, the association between low television watching and boys’ self-harm should be interpreted with caution. Further confirmatory studies are needed to clarify this issue.

Our television ST results are inconsistent with some previous studies reporting a general negative (Chen and Lu, 2009) or null (Hong et al., 2009) association with mental health parameters. The differences may be related to the fact that we compared multiple exposure volumes, rather than only two (<2 vs. ≥2 h per day) or rather than treating television exposure as a continuous variable. Additionally, we examined ST for weekdays only, while other researchers have averaged weekday and weekend ST.

Although, we supposed that associations with psychiatric problems and self-harm may be stronger for VG/CU than for television, it was still unexpected that even the smallest exposure volume of VG/CU in a typical school day would be associated with self-harm and with psychiatric problems, with the exceptions of anxiety and ADHPs in male adolescents. These findings were consistent with some previous studies, despite methodological differences such as having only two (e.g., <2 vs. ≥2 h per day) ST levels (Desai et al., 2010; Cao et al., 2011). Conversely, these results conflict with prior studies suggesting a U-shaped ST dose-response curve (Bélanger et al., 2011; Kim, 2012). For example, Bélanger et al. (2011) found evidence of a possible “U”-shaped association between internet use and depression risk in adolescents. Specifically, they found that non-internet (male or female) or occasional internet (female) users were at greater risk of depression than regular internet users. However, these studies estimated internet ST by averaging weekday and weekend exposure volumes. As mentioned above, adolescents spend much more time on media screens on weekend days than on weekdays (Biddle et al., 2009). Therefore, such non-discriminating averages may obscure an association between VG/CU exposure in a typical school day and the risks of psychiatric problems and self-harm.

Given the aforementioned difficulty in comparing our results with those of studies that averaged weekday and weekend ST exposure data, it is quite interesting to compare the present study to a methodologically more similar study conducted (Messias et al., 2011) in the USA. They found that only more than 3 h per school day VG/CU exposure was associated with higher suicidal ideation in a 2007 sample, and then observed a “U”-shaped association between VG/CU exposure volumes and suicide planning and suicide attempt in a 2009 sample. Hence, there may be socio-cultural differences in VG/CU association with psychiatric symptoms (Greenberger et al., 2000). Relative to their counterparts in the USA, kids in mainland China spend more time immersed in schoolwork and are, generally, under greater competitive pressure in their academics. Chinese secondary school pupils have longer school days (∼12 h/day) than their counterparts in most developed Western countries. Even when they are not at school, they are expected by their parents to attend extra classes and have substantial homework expectations. Consequently, children in China have much less time for non-academic activities. This circumstance is reflected by the observation that majorities of the participants in our study reported having no television (58.6%) and no VG/CU (70.2%) ST on typical school days. By contrast, relatively few American kids (18.5%) had no VG/CU exposure on typical weekdays (Messias et al., 2011). It could be that VG/CU exposure has a more potent negative influence on adolescents’ mental health in China than in the USA because Chinese children may have reduced exposure to protective factors, such as physical activity and sufficient sleep, as well as greater stress related to homework time demands and academic competitiveness, which may elevate their vulnerability to mental health problems (Georgiou et al., 2015; Liu et al., 2015a), including risk of suicide (Liu, 2004).

Stratification of our results by screen type revealed different associations with psychiatric problems and self-harm between television versus VG/CU exposure, with the latter being much stronger than the former, consistent with several previous studies (Hong et al., 2009; Desai et al., 2010; Messias et al., 2011; Kim, 2012). It could be that VG/CU content is less supervised and may contain more potentially unhealthy or negative influences. Regardless, it is worth mentioning that prior studies that have differentiated ST type have been overall quite inconsistent. Godinho et al. (2014) found no significant association of television or computer ST with depressive symptoms in male adolescents. Casiano et al. (2012) found no effect of television ST and an inverse relationship between video game playing time and depressive risk in adolescents. And in a longitudinal cohort study (Primack et al., 2009) showed a significant association between television ST (in the last week), but not video game playing time, and elevated risk of subsequent depression symptoms. All three of these studies, unlike the present study, averaged weekday and weekend exposures. Additionally, these studies were conducted in Portugal, Canada, and the USA, respectively, which are all developed countries with greater in-home VG/CU accessibly than in mainland China, where most teenagers have to leave their homes and go to internet cafes for VG/CU access, a habit which has been reported to be a risk factor affecting psychological health in high school students (Zhang et al., 2011). Hence, different recommendations concerning ST limitations may be needed in different countries or regions. Limitation guidelines should perhaps also differ between school and weekend days.

This study reported associations between multiple ST volumes in a typical school day and adolescents’ risks of psychiatric problems and self-harm in a large representative mainland Chinese sample. These findings are informative for parents, educators, and pediatricians in mainland China, as well as in other similar developing countries or regions where adolescents’ ST exposure is not yet highly prevalent. Notwithstanding, this study has several limitations. Firstly, as in all cross-sectional studies, the observed associations may be influenced by other health-risk behaviors (He et al., 2006) beyond the controlled-for variables. Furthermore, we cannot confirm direction of causality. For example, it is possible that (already) depressed adolescents choose to spend more time engaged in VG/CU than non-depressed adolescents, rather than VG/CU time being a causative factor. Secondly, we did not examine ST exposure on weekend days. Given the inconsistencies between our results and several other studies that averaged weekday and weekend data together, it may be necessary to analyze *both* weekday and weekend ST, and to do so *separately*, which has yet to be done. In this regard, caution should be exercised when explaining findings in this study, especially for VG/CU time, because although any VG/CU exposure in a typical school day was associated with greater risk of psychiatric problems and self-harm in mainland Chinese adolescents, VG/CU exposure during the weekend may not be. Considering the findings from the current study are exploratory results, further confirmatory studies are needed to test the corresponding hypotheses. Also, the findings in this study may not generalize to juveniles who do not go to school, such as juveniles who are homeschooled or juveniles who are truant from school. Finally, screen type categories in this study did not include tablets or smart phones. Future studies should incorporate data for these new screen types.

## Conclusion

For mainland Chinese high school students, excessive television exposure (>2 h) in a typical school day was associated with higher risk of depression, anxiety, GEBSPs, and ODPs. However, >0 to ≤1 1 h per school day of television exposure appeared to be associated with lower risk of self-harm in male adolescents. Meanwhile, even very limited regular school-day VG/CU exposure was found to be associated with higher risk of psychiatric problems and self-harm in both genders. These findings highlight the potential role of school-day ST, especially VG/CU time, in the primary prevention of adolescent psychiatric problems and self-harm.

## Author Contributions

SY and JY contributed to the conception and design of the study. ML and QM were involved in data collection and analysis, and all authors interpreted the results. ML drafted the manuscript, and all authors revised and approved the version to be published.

## Conflict of Interest Statement

The authors declare that the research was conducted in the absence of any commercial or financial relationships that could be construed as a potential conflict of interest.

## Abbreviations

ADHPs: attention deficit/hyperactivity problems
BMI: body mass index
CPs: conduct problems
ODPs: oppositional defiant problems
ST: screen time
VG/CU: playing video or computer games or using a computer for non-educational purpose

## References

[B1] Achenbach T. M. Dumenci L. Rescorla L. A. (2001). *Ratings of Relations Between DSM-IV Diagnostic Categories and Items of the CBCL/6-18, TRF, and YSR*. Burlington, VT: University of Vermont.

[B2] Achenbach T. M. Rescorla L. (2001). *ASEBA School-Age Forms & Profiles.* Burlington: Aseba.

[B3] Bandura A. (2001). Social cognitive theory of mass communication. *Media Psychol.* 3 265–299. 10.1207/S1532785XMEP0303_03

[B4] Bélanger R. E. Akre C. Berchtold A. Michaud P.-A. (2011). A U-shaped association between intensity of Internet use and adolescent health. *Pediatrics* 127 e330–e335. 10.1542/peds.2010-1235 21242218

[B5] Biddle S. J. Gorely T. Marshall S. J. Cameron N. (2009). The prevalence of sedentary behavior and physical activity in leisure time: a study of Scottish adolescents using ecological momentary assessment. *Prev. Med.* 48 151–155. 10.1016/j.ypmed.2008.10.025 19046984

[B6] Blum R. W. Bastos F. I. Kabiru C. W. Le L. C. (2012). Adolescent health in the 21st century. *Lancet* 379 1567–1568. 10.1016/S0140-6736(12)60407-3 22538177

[B7] Borowsky I. W. Ireland M. Resnick M. D. (2001). Adolescent suicide attempts: risks and protectors. *Pediatrics* 107 485–493. 10.1542/peds.107.3.485 11230587

[B8] Brener N. D. Kann L. McManus T. Kinchen S. A. Sundberg E. C. Ross J. G. (2002). Reliability of the 1999 youth risk behavior survey questionnaire. *J. Adolesc. Health* 31 336–342. 10.1016/S1054-139X(02)00339-7 12359379

[B9] Cao H. Qian Q. Weng T. Yuan C. Sun Y. Wang H. (2011). Screen time, physical activity and mental health among urban adolescents in China. *Prev. Med.* 53 316–320. 10.1016/j.ypmed.2011.09.002 21933680

[B10] Carli V. Hoven C. W. Wasserman C. Chiesa F. Guffanti G. Sarchiapone M. (2014). A newly identified group of adolescents at “invisible” risk for psychopathology and suicidal behavior: findings from the SEYLE study. *World Psychiatry* 13 78–86. 10.1002/wps.20088 24497256 PMC3918027

[B11] Casiano H. Kinley D. J. Katz L. Y. Chartier M. J. Sareen J. (2012). Media use and health outcomes in adolescents: findings from a nationally r. *J. Can. Acad. Child Adolesc. Psychiatry* 21 296–301. 23133464 PMC3490531

[B12] Chen S. Y. Lu L. (2009). After-school time use in Taiwan: effects on educational achievement and well-being. *Adolescence* 44 891–909. 20432606

[B13] Coyne S. M. Padilla-Walker L. M. Fraser A. M. Fellows K. Day R. D. (2014). Media Time = family time” positive media use in families with adolescents. *J. Adolesc. Res.* 29 663–688. 10.1177/0743558414538316

[B14] Desai R. A. Krishnan-Sarin S. Cavallo D. Potenza M. N. (2010). Video-gaming among high school students: health correlates, gender differences, and problematic gaming. *Pediatrics* 126 e1414–e1424. 10.1542/peds.2009-2706 21078729 PMC3678538

[B15] Eggermont S. Van den Bulck J. (2006). Nodding off or switching off? The use of popular media as a sleep aid in secondary-school children. *J. Paediatr. Child Health* 42 428–433. 10.1111/j.1440-1754.2006.00892.x 16898880

[B16] Georgiou E. Matthias E. Kobel S. Kettner S. Dreyhaupt J. Steinacker J. M. (2015). Interaction of physical activity and interoception in children. *Front. Psychol.* 6:502 10.3389/fpsyg.2015.00502 PMC4411994 25972827

[B17] Godinho J. Araujo J. Barros H. Ramos E. (2014). Characteristics associated with media use in early adolescence. *Cad. Saude. Publica* 30 587–598. 10.1590/0102-311x00100313 24714948

[B18] Greenberger E. Chen C. Tally S. R. Dong Q. (2000). Family, peer, and individual correlates of depressive symptomatology among US and Chinese adolescents. *J. Consult. Clin. Psychol.* 68:209 10.1037/0022-006X.68.2.209 10780120

[B19] Haarasilta L. M. Marttunen M. J. Kaprio J. A. Aro H. M. (2004). Correlates of depression in a representative nationwide sample of adolescents (15–19 years) and young adults (20–24 years). *Eur. J. Public Health* 14 280–285. 10.1093/eurpub/14.3.280 15369034

[B20] Hamar P. Biddle S. Soós I. Takács B. Huszár A. (2010). The prevalence of sedentary behaviours and physical activity in Hungarian youth. *Eur. J. Public Health* 20 85–90. 10.1093/eurpub/ckp100 19587226

[B21] He F. J. Nowson C. A. MacGregor G. A. (2006). Fruit and vegetable consumption and stroke: meta-analysis of cohort studies. *Lancet* 367 320–326. 10.1016/S0140-6736(06)68069-0 16443039

[B22] Hong X. Li J. Xu F. Tse L. A. Liang Y. Wang Z. (2009). Physical activity inversely associated with the presence of depression among urban adolescents in regional China. *BMC Public Health* 9:148 10.1186/1471-2458-9-148 PMC2693135 19457241

[B23] Kessler R. C. Angermeyer M. Anthony J. C. De Graaf R. Demyttenaere K. Gasquet I. (2007). Lifetime prevalence and age-of-onset distributions of mental disorders in the World Health Organization’s World Mental Health Survey Initiative. *World Psychiatry* 6:168. PMC2174588 18188442

[B24] Kieling C. Baker-Henningham H. Belfer M. Conti G. Ertem I. Omigbodun O. (2011). Child and adolescent mental health worldwide: evidence for action. *Lancet* 378 1515–1525. 10.1016/S0140-6736(11)60827-1 22008427

[B25] Kim J. Y. (2012). The nonlinear association between Internet using time for non-educational purposes and adolescent health. *J. Prev. Med. Public Health* 45 37–46. 10.3961/jpmph.2012.45.1.37 22389757 PMC3278603

[B26] Kim K. Ryu E. Chon M.-Y. Yeun E.-J. Choi S.-Y. Seo J.-S. (2006). Internet addiction in Korean adolescents and its relation to depression and suicidal ideation: a questionnaire survey. *Int. J. Nurs. Stud.* 43 185–192. 10.1016/j.ijnurstu.2005.02.005 16427966

[B27] Kim-Cohen J. Caspi A. Moffitt T. E. Harrington H. Milne B. J. Poulton R. (2003). Prior juvenile diagnoses in adults with mental disorder: developmental follow-back of a prospective-longitudinal cohort. *Arch. Gen. Psychiatry* 60 709–717. 10.1001/archpsyc.60.7.709 12860775

[B28] Kraut R. Patterson M. Lundmark V. Kiesler S. Mukophadhyay T. Scherlis W. (1998). Internet paradox: a social technology that reduces social involvement and psychological well-being? *Am. Psychol.* 53:1017 10.1037/0003-066X.53.9.1017 9841579

[B29] Liu M. Wu L. Ming Q. (2015a). How does physical activity intervention improve self-esteem and self-concept in children and adolescents? Evidence from a meta-analysis. *PLoS ONE* 10:e0134804 10.1371/journal.pone.0134804 PMC4524727 26241879

[B30] Liu M. Wu L. Yao S. (2015b). Dose response association of screen time-based sedentary behavior in children and adolescents and depression: a meta-analysis of observational studies. *Br. J. Sports Med.* 10.1136/bjsports-2015-095084 [Epub ahead of print]. PMC4977203 26552416

[B31] Liu X. (2004). Sleep and adolescent suicidal behavior. *Sleep* 27 1351–1358. 15586788 10.1093/sleep/27.7.1351

[B32] March J. S. Parker J. D. Sullivan K. Stallings P. Conners C. K. (1997). The Multidimensional Anxiety Scale for Children (MASC): factor structure, reliability, and validity. *J. Am. Acad. Child Adolesc. Psychiatry* 36 554–565. 10.1097/00004583-199704000-00019 9100431

[B33] McHale S. M. Crouter A. C. Tucker C. J. (2001). Free-time activities in middle childhood: links with adjustment in early adolescence. *Child Dev.* 72 1764–1778. 10.1111/1467-8624.00377 11768144

[B34] Messias E. Castro J. Saini A. Usman M. Peeples D. (2011). Sadness, suicide, and their association with video game and internet overuse among teens: results from the youth risk behavior survey 2007 and 2009. *Suicide Life Threat Behav.* 41 307–315. 10.1111/j.1943-278X.2011.00030.x 21463355

[B35] Padilla-Walker L. M. Coyne S. M. Fraser A. M. (2012). Getting a high-speed family connection: associations between family media use and family connection. *Fam Relat.* 61 426–440. 10.1111/j.1741-3729.2012.00710.x

[B36] Patel V. Flisher A. J. Hetrick S. McGorry P. (2007). Mental health of young people: a global public-health challenge. *Lancet* 369 1302–1313. 10.1016/S0140-6736(07)60368-7 17434406

[B37] Pediatrics A. A. O. (2001). American Academy of Pediatrics: Children, adolescents, and television. *Pediatrics* 107:423 10.1542/peds.107.2.423 11158483

[B38] Primack B. A. Swanier B. Georgiopoulos A. M. Land S. R. Fine M. J. (2009). Association between media use in adolescence and depression in young adulthood: a longitudinal study. *Arch. Gen. Psychiat.* 66 181–188. 10.1001/archgenpsychiatry.2008.532 19188540 PMC3004674

[B39] Rideout V. J. Foehr U. G. Roberts D. F. (2010). *Generation M [superscript 2]: Media in the Lives of 8-to 18-Year-Olds*. Menlo Park, CA: Henry J. Kaiser Family Foundation.

[B40] Roberts R. E. Lewinsohn P. M. Seeley J. R. (1991). Screening for adolescent depression: a comparison of depression scales. *J. Am. Acad. Child Adolesc. Psychiatry* 30 58–66. 10.1097/00004583-199101000-00009 2005065

[B41] Shapira N. A. Goldsmith T. D. Keck P. E. Khosla U. M. McElroy S. L. (2000). Psychiatric features of individuals with problematic internet use. *J. Affect. Disord.* 57 267–272. 10.1016/S0165-0327(99)00107-X 10708842

[B42] Strasburger V. C. Jordan A. B. Donnerstein E. (2010). Health effects of media on children and adolescents. *Pediatrics* 125 756–767. 10.1542/peds.2009-2563 20194281

[B43] Tremblay M. S. LeBlanc A. G. Kho M. E. Saunders T. J. Larouche R. Colley R. C. (2011). Systematic review of sedentary behaviour and health indicators in school-aged children and youth. *Int. J. Behav. Nutr. Phys. Act.* 8:98 10.1186/1479-5868-8-98 PMC3186735 21936895

[B44] Van den Bulck J. (2004). Television viewing, computer game playing, and Internet use and self-reported time to bed and time out of bed in secondary-school children. *Sleep* 27 101–104. 14998244 10.1093/sleep/27.1.101

[B45] Wang M. Armour C. Wu Y. Ren F. Zhu X. Yao S. (2013). Factor Structure of the CES-D and measurement invariance across gender in mainland chinese adolescents. *J. Clin. Psychol.* 69 966–979. 10.1002/jclp.21978 23775279

[B46] Wang M. Yi J. Cai L. Hu M. Zhu X. Yao S. (2012). Development and psychometric properties of the health-risk behavior inventory for Chinese adolescents. *BMC Med. Res. Method* 12:94 10.1186/1471-2288-12-94 PMC3431231 22770389

[B47] Yao S. Zhang C. Zhu X. Jing X. McWhinnie C. M. Abela J. R. (2009). Measuring adolescent psychopathology: psychometric properties of the self-report strengths and difficulties questionnaire in a sample of Chinese adolescents. *J. Adolesc. Health* 45 55–62. 10.1016/j.jadohealth.2008.11.006 19541250

[B48] Yao S. Zou T. Zhu X. Abela J. R. Auerbach R. P. Tong X. (2007). Reliability and validity of the Chinese version of the Multidimensional Anxiety Scale for Children among Chinese secondary school students. *Child Psychiatry Hum. Dev.* 38 1–16. 10.1007/s10578-006-0039-0 17109221

[B49] Zhang Y. Xu J. Liu S. Zeng L. Cheng J. Yang M. H. (2011). Investigation on epidemiology of mental health and its related factors among 2474 high school students in dongguan of guangdong. *J. Ningxia Med. Univ.* 33 840–844.

